# Structure Determination of the Nuclear Pore Complex with Three-Dimensional Cryo electron Microscopy

**DOI:** 10.1016/j.jmb.2016.01.004

**Published:** 2016-05-22

**Authors:** Alexander von Appen, Martin Beck

**Affiliations:** European Molecular Biology Laboratory, Structural and Computational Biology Unit, Cell Biology and Biophysics Unit, Meyerhofstraße 1, 69117 Heidelberg, Germany

**Keywords:** NPC, nuclear pore complex, NE, nuclear envelope, EM, electron microscopy, 3D, three-dimensional, cryoEM, cryoelectron microscopy, RCT, random conical tilt, *X.l.*, *Xenopus laevis*, *S.c.*, *Saccharomyces cerevisiae*, cryoET, cryoelectron tomography, *D.d.*, *Dictyostelium discoideum*, nuclear pore complex, cryoelectron microscopy

## Abstract

Determining the structure of the nuclear pore complex (NPC) imposes an enormous challenge due to its size, intricate composition and membrane-embedded nature. In vertebrates, about 1000 protein building blocks assemble into a 110-MDa complex that fuses the inner and outer membranes of a cell's nucleus. Here, we review the recent progress in understanding the *in situ* architecture of the NPC with a specific focus on approaches using three-dimensional cryo electron microscopy. We discuss technological benefits and limitations and give an outlook toward obtaining a high-resolution structure of the NPC.

## Highlights

• Overview over three-dimensional electron microscopic analysis of the nuclear pore complex.
• Review of recent integrative structural biology studies of the nuclear pore complex scaffold architecture.

## Graphical Abstract

**Figure:**
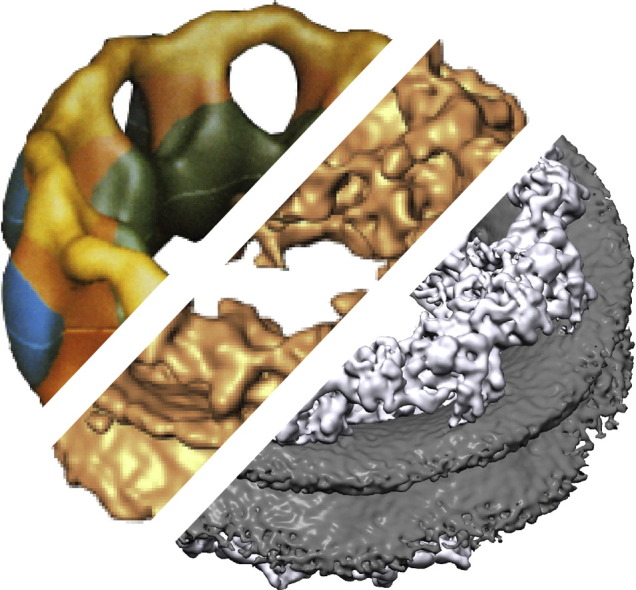

## Composition of Nuclear Pore Complexes

Nuclear pores direct the transport of biomolecules across the nuclear envelope (NE). Morphologically, nuclear pore complexes (NPCs) contain three stacked rings. The inner ring spans the fused inner and outer nuclear membranes. The cytoplasmic and a nucleoplasmic rings sandwich the inner ring from both distal ends [1]. The protein components constituting this structure have been relatively well characterized. NPCs assemble from multiple copies of ~ 30 nucleoporins (Nups) that underlie a certain modularity [2], [3]: individual protein building blocks are preassembled into subcomplexes that subsequently join each other in multiple copies to form the large NPC structure.

Two of these subcomplexes, the so-called Y and the inner ring complexes, constitute the NPC scaffold. This core structure consists primarily of alpha solenoid and beta propeller domains. The Y-complex is the structurally best-defined subcomplex and displays a characteristic Y-shape. Both the Y-shaped outline [4], [5], [6], [7] and the six core proteins constituting it are conserved throughout eukaryotes [8]. The human Y-complex contains four auxiliary beta propellers, namely, Nup43, Nup37, Seh1 and Elys, whereby the latter contains additional domain features and binds specifically to the nuclear face of the NPC [9], [10]. The Y-complex assembles into a vertex consisting of a small arm, a large arm and a stem base that join each other in the center of the Y. The small arm of the Y contains Nup85 and Seh1 (in higher eukaryotes, also Nup43); the large arm contains Elys, Nup37 and Nup160. The stem base consists of Nup96 and Sec13, which joins Nup85 and Nup160 in the central hub element. The highly flexible stem tip contains Nup133 and Nup107 [7], [8]. The inner ring complex contains five members in vertebrates, namely, Nup205, Nup188, Nup155, Nup93 and Nup53 [11]. Its actual outline is less well understood (see below).

Several other subcomplexes bind peripherally to the scaffold and are important for the interaction with cargo and other NPC functions. The trimeric Nup62–Nup58–Nup54 subcomplex localizes symmetrically to the central channel region of the NPC [12], [13], [14]. Other subcomplexes asymmetrically bind either the nuclear face or the cytoplasmic face of the NPC. The Nup214–Nup88–Nup62 subcomplex that also contains Rae1 and Nup98 residues on the cytoplasmic side [15], [16] and is essential for mRNA export [17]. Also, the Nup358–RanGAP1*SUMO1–Ubc9 subcomplex [18] is associated to the cytoplasmic ring and regulates the nuclear transport receptor–cargo complex assembly and disassembly [19]. The exact interfaces of the remaining components are less well understood. Tpr, Nup50 and Nup153 bind to the Y-complex specifically on the nucleoplasmic side [20]. Only three Nups, Pom121, gp210 and Ndc1, are integral membrane proteins in vertebrates [20], [21] and cross the lipid bilayer of the NE with a single alpha helix (Pom121 and gp210) or with a larger transmembrane domain (Ndc1). While cell-type specifically expressed Pom121 appears to bind to both the Y and inner ring complexes [22], the ubiquitous Ndc1 binds the inner ring complex [23]. Only Ndc1 appears to be conserved throughout eukaryotes.

## Investigation of the NPC Structure by Classic Electron Microscopy Techniques

NPCs have been discovered using electron microscopy (EM) already in the 1950s [24]. Their size, obvious topology and specific localization to the NE have since then rendered NPCs as easy targets for classic EM. Many studies used transmission EM of plastic-embedded sections and scanning EM in combination with rotary heavy-metal shadowing to investigate nuclear pore structure, morphology and function. These studies have strongly influenced the current scientific concepts of the nucleocytoplasmic transport system. That is, they revealed that cellular material passes through a central channel of the NPC, defined the size limits for its cargos (39 nm), discovered the general morphological features of NPCs and allowed localizing candidate protein components to the NPC and its subdomains (see, e.g., Refs. [1], [25], [26], [27], [28], [29], [30]). However, all of these classical EM techniques suffer from specific limitations. The heavy-metal shadowing and staining techniques might overemphasize the observed structures or be more susceptible for specific subdomains than others. The two-dimensional imaging procedures considerably limit the attainable resolution and thus restrict the biological questions that can be addressed. Three-dimensional (3D) reconstruction techniques [31] and cryoelectron microscopy (cryoEM) of near-native, frozen-embedded samples [32], [33] that contain NPCs in an *in situ* scenario have meanwhile proven their potential to overcome some of these limitations.

## 3D Reconstructions of the NPC

**Fig. 1:**
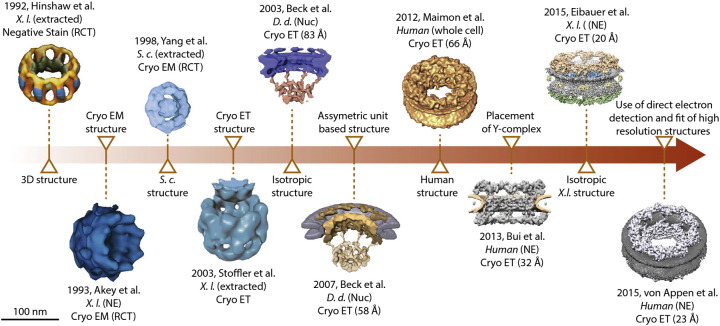
3D structures of the NPC that were solved using EM. The timeline shows representative structures of the NPC drawn to scale. The model organism, the method used and the reported resolution are indicated when applicable. Images have been modified from the cited references (extracted: NPCs were extracted from membranes using heparin and/or detergent; Nuc: NPCs were embedded in the NE of intact nuclei; NE: NPCs were embedded into isolated NEs; whole cell: data were acquired on the intact cells).

**Fig. 2:**
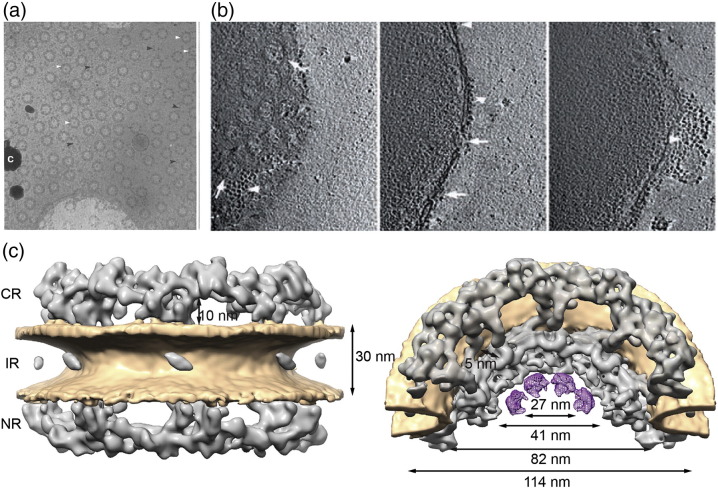
Overview of the NPC scaffold architecture and biological specimen used for its structure determination [39]. (a) Cryoelectron micrograph of spread *X.l.* oocyte with high NPC density [36]. (b) Three sequential *x*–*y* slices of 10 nm in thickness through a tomogram of a *D.d.* nucleus. Arrows indicate NPC in top view (left) and side view (right). Arrowheads show ribosomes decorating the outer nuclear membrane. (c) Isosurface rendering of the structure of the human NPC resolved to 33 Å seen from front (left), cut in half and tilted (right). Membranes are shown in brown. Dimensions of CR, IR and NR are indicated (adapted from Ref. [7]).

Hinshaw *et al*. obtained the first 3D reconstruction of the NPC by applying the random conical tilt (RCT) method to negatively stained *Xenopus laevis* (*X.l.*) NE spreads on EM grids [34], which contain a large number of NPCs per surface area (Fig. 1, Fig. 2a). The RCT method is based on the acquisition of electron micrographs at different tilt angles. These projections are subsequently used to reconstruct a 3D map of the specimen [35]. The first cryoEM reconstructions were obtained of the *X.l.* [36] and *Saccharomyces cerevisiae* (*S.c.*) NPCs [37] using the RCT method. In the following, also cryoelectron tomography (cryoET) was used to 3D reconstruct the *X.l.* NPC [38]. In contrast to RCT, the tomographic approach is less error prone because it more comprehensively acquires projections at all possible tilts and subsequently combines subtomograms that contain individual NPCs by iterative averaging. However, all the above-mentioned studies sampled the NPCs primarily or exclusively in top view, which orients the nucleocytoplasmic axes of the NPCs parallel with the electron optical axis (Fig. 2a). Since specimen holders in transmission electron microscopes tilt only up to ~ 60°, the resulting reconstructions lacked isotropic angular coverage. If none of the primary images contained NPCs in side view so that the fused inner (INM) and outer (ONM) nuclear membranes are visible, these features will also not be resolved in the resulting reconstruction. Nevertheless, these early reconstructions contained features that resembled the 8-fold rotationally symmetry of the NPC and captured the rough overall dimensions.

The angular coverage problem was first addressed by a study that conducted cryoET analyses of nuclei isolated from the lower eukaryote *Dictyostelium discoideum* (*D.d.*) [39]. The NE was not spread on the EM grid but embedded with its curvature to enable imaging of NPC in all possible orientations, including side views in which the membrane is visible (compare Fig. 2a and b). After tomographic reconstruction of the NE, subtomograms that contain individual NPCs seen in various different orientations are extracted *in silico*. Although each individual subtomogram has a nonisotropic angular coverage due to the tilt restriction of the microscope, the combination of many covers all necessary projection angles. In this way, subtomogram averaging allows us to obtain an isotropic 3D reconstruction. With an overall resolution of 8–9 nm, the *D.d.* NPC structure for the first time resolved the fused membranes and the three stacked rings. Numerous tomographic studies have since then used this so-called “missing wedge weighted subtomogram averaging procedure” [39], [40] to analyze the structure of various protein complexes, often in the context of membranes *in situ* (for review, see, e.g., Ref. [41]).

The intrinsic structural plasticity of the NPC [3] had to be taken into account in order to further improve the resolution. Subtomogram averaging focused on the asymmetric units instead of entire NPCs, that is, the single elements of the 8-fold rotational assembly, corrected for deviations from the ideal symmetry (so-called symmetry-independent averaging). This strategy facilitated improving the resolution of the NPC structure to 58 Å [42]. The resulting reconstruction revealed that the NPC consisted of a symmetric inner ring structure, while the distal cytoplasmic and nucleoplasmic rings show certain differences likely because different subcomplexes bind to them. The same computational structure determination framework was also used to obtain the first reconstruction of the human NPC. This was experimentally very challenging because human tissue culture cells contain much fewer NPCs per surface area as compared to other experimental model systems [39], [43], [44]. Maimon *et al.* plunge froze U2OS cells directly grown on EM grids and subjected them to cryoET analysis. Since U2OS cells have a spread-out morphology, the NPC could be directly imaged in the cell. This reduces artifacts that are potentially induced during the sample preparation to a minimum [45]. This technique required the sample to be embedded in relatively thick ice, which limited the signal-to-noise ratio of the primary data and, as a consequence, the overall resolution. Nevertheless, Maimon *et al.* isotropically resolved the human NPC structure to 66 Å and revealed that its overall dimensions are 120 nm in diameter and 85 nm in height along the transport axis.

The above-discussed cryoEM studies of the human, *D.d.*, *X.l.* and *S.c.* NPCs revealed, at first glance, striking structural differences, most notably in the overall size and the separation of the NPC subdomains, for example, the different diameters of the three rings (Fig. 1). However, since the early studies are based on nonisotropic reconstructions, structural features appear elongated and distorted along the electron optical axis (identical with the nucleocytoplasmic axis). As a consequence, the structures can be objectively compared neither to each other nor to the isotropic reconstructions of the human and *D.d.* NPCs. Some of the aforementioned studies also included membrane extraction steps to enrich for nuclear pores [34], [37], [46] and thus did not necessarily investigate a structural state that reflects an *in situ* scenario. Since in the technical standards for measuring the resolution of cryoEM structures became more rigorous over the years [47], [48], a meaningful comparison of the reported resolution is not straightforward. Nevertheless, if the isotropic reconstructions of the human and *D.d.* NPCs are compared, their overall size and basic features are astonishingly similar.

## Reconstructions of the NPC in the 2-nm-Resolution Regime

**Fig. 3:**
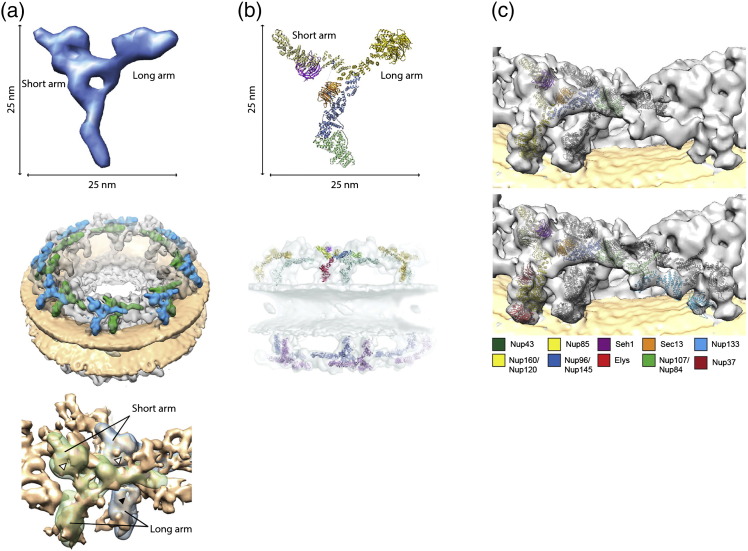
The Y-complex assembles into two reticulated concentric rings within the nuclear and cytoplasmic rings of the NPC. (a) Negative staining structure of the human Y-complex at 33 Å resolution (top) that was localized by a systematic fitting approach within the tomographic map of the human NPC (32 Å resolution; middle) and the *X.l.* NPC structure (20 Å resolution; bottom). Images reproduced from Refs. [7], [50]. (b) Crystal structure of the Y-complex vertex [51], [53] (top) that was localized within the tomographic map of the human NPC resolved to 32 Å [7] (bottom). (c) Fits of X-ray structures of sufficient size into the tomographic map of the human NPC resolved to 23 Å [51]. The fit of the X-ray structure of the yeast vertex (top [53]) is shown in comparison to an independently obtained hybrid model of the entire Y-complex (bottom [51]).

The NPC assembles in a modular manner from subcomplexes. This phenomenon might be exploited to localize subcomplexes. If EM maps are obtained of both, the entire NPC and isolated subcomplexes, those might be systematically compared. The first 3D EM map of the NPC that was sufficiently resolved to correlate its density with the structural signatures of isolated subcomplexes was obtained by cryoET applied to NEs, which were isolated from human tissue culture cells [7], [49]. Due to the batch preparation procedure, a larger data set with better signal-to-noise ratio was obtained which resolved the NPC structure to ~ 33 Å [7] (Fig. 2c). At this resolution, features within the tomographic map displayed obvious similarities to the isolated human Y-complex: a systematic search for the structural signature of a 3D negative staining structure of the isolated human Y-complex vertex revealed for the first time how 32 copies of it assemble into two concentric, slightly shifted rings on both cytoplasmic and nucleoplasmic sites to form the NPC scaffold (Fig. 3) [7].

Two recently published tomographic maps of the *X.l.* NPC structure by Eibauer *et al.* [50] (Fig. 3a) and the human NPC [51] (Fig. 3c), the latter one based on direct electron detection [52], were reported with a resolution in the 2-nm regime. The tomographic map of the *X.l.* NPC is the first isotropic structure from this organism and addressed the angular coverage problem by folding the *X.l.* NEs on the EM grid [50]. Both systems have advantages and disadvantages. While *X.l.* oocyte NEs contain more NPCs per surface area and are potentially well suited for investigating the interaction of cargos with the NPC, the human system is easier to genetically manipulate in order to perform perturbation experiments, as exemplified by the gene silencing experiments that revealed the localization of the Nup214 complex to a protrusion of the CR and the scaffolding function of Nup358 [7], [51]. A comparison of the human and the *X.l.* structure reveals very similar overall dimensions that are in line with previous reconstructions [7], [39], [42], [45]. The Y-complex double-ring signature is clearly visible in the CR of both reconstructions (Fig. 3a). Although the symmetry of NPC scaffold components across the NE plane has been firmly established [20], the Y-complex signature is not as obvious in the NR of the *X.l.* structure. It will thus be very interesting to see if those differences manifest in future reconstructions of the *X.l.* NPC.

## Integration of Tomographic Maps with X-ray Structures and Other Complementary Data

Continuous efforts in X-ray crystallography have revealed the high-resolution structure of the majority of the NPC scaffold Nups [20]. Recently, two crystal structures covering the last missing part of the Y-complex, the central hub element of vertex region, were published. Stuwe *et al.* analyzed the *S.c.* Y-complex vertex as a whole and engineered an antibody to stabilize the structure. The dimensions of the crystal structure are similar to the structure of the human Y-complex vertex determined by negative-stain electron tomography (Fig. 3a and b). A systematic fitting approach was used to analyze the tomographic map of the human NPC for structural similarity [53] and confirmed the Y-complex double-ring assembly proposed by Bui *et al.* [7]. The second study by Kelly *et al.* focused on a smaller fragment covering the central hub element of the Y-complex complex from the eukaryotic thermophile *Myceliophthora thermophila*. In order to build a full atomic model of the Y-complex, a composite structure was generated based on overlapping segments and by modeling an 84-amino-acid segment of Nup107 that resides to the stem region of the Y-complex [8]. Here, a comparison of the structural similarity between the rigid Y-complex composite model and the tomographic map of the human NPC provided by Bui *et al.* resulted in sterical clashes in the stem region of the two stacked Y-complexes. However, Bui *et al.* also showed that the isolated human Y-complex is very flexible specifically in the stem region and can therefore assume multiple confirmations [7]. Interestingly, the structures from Stuwe *et al.* and Kelley *et al.* have considerably different conformations, which might hint at intrinsic flexibility of the Y-complex but might also be attributed to interspecies differences.

This issue is resolved when X-ray structures are fitted into the meanwhile available higher-resolved tomographic map [51] because the flexible stem region of the Y-complex, in particular, the shape of Nup133, becomes much more obvious (Fig. 3c). This analysis revealed that the *in situ* oligomerization of the Y-complex requires at least five intersubcomplex interfaces, including the head-to-tail interaction of Nup160 with Nup133 across subunits [54], [55], which facilitates the ring formation of the NPC. Both the inner and outer rings form a closed entity on their own [51]. The flexible stem region of the Y-complex and the attachment of the terminal beta propeller of Nup133 thereby establish the two rings with slightly different diameters and circumference. Surprisingly, one of the Y-complex interfaces, an alpha helical protrusion of the inner Nup107 called finger domain [56], with the outer Nup43 are both absent in *S.c.* (see Ref. [51] for detail) indicating that this contact has no relevance in *S.c.* NPC structure. This finding points to possible architectural or oligomeric differences between organisms that await further investigation.

Several recent studies used systematic fitting approaches that systematically explore the tomographic maps for similarity with X-ray structures to determine the location and orientation of substructures [7], [8], [51], [53]. Since the majority of the NPC scaffold is composed of alpha solenoid and beta propeller folds, the shape of different Nups might appear as similar at the current 2-nm resolution of the tomographic maps. Also, potential flexibility of the protein structure needs to be considered. The integration with complementary experimental data such as, for example, proximity information, stoichiometry, biochemical data or correlation of EM data with superresolution microscopy is required to independently confirm the fits [7], [49], [57], [58]. In case of the Y-complex localization within the NPC scaffold, additional data that confirm the assignment are available. Mass spectrometric measurements of Nup stoichiometry [49] and superresolution light microscopy measurements determined the copy number and the orientation of the Y-complex *in situ* [49], [59]. It has also turned out consistent with further structural studies, namely, a mass spectrometric study that used proximity labeling to probe Y-complex arrangement *in vivo*, biochemical data demonstrating the head-to-tail interaction of Y-complex members, use of immunoelectron microscopy demonstrating *C*2 symmetric localization of the Y-complex [26], [54], [60] and removal of the outer Y-complex upon gene silencing of Nup358 [51]. Superresolution light microscopy measurements have also revealed insights into the more dynamic, peripheral components of the NPC, such as, for example, the approximate distance of gp210 from the central axis [57], [61]. Although those dynamic features do often not manifest in cryoEM averages, the position of gp210 might be in line with relatively featureless density observed in the lumen of both nuclear membranes (Fig. 2c).

## A First Glimpse onto the Inner Ring Architecture

**Fig. 4:**
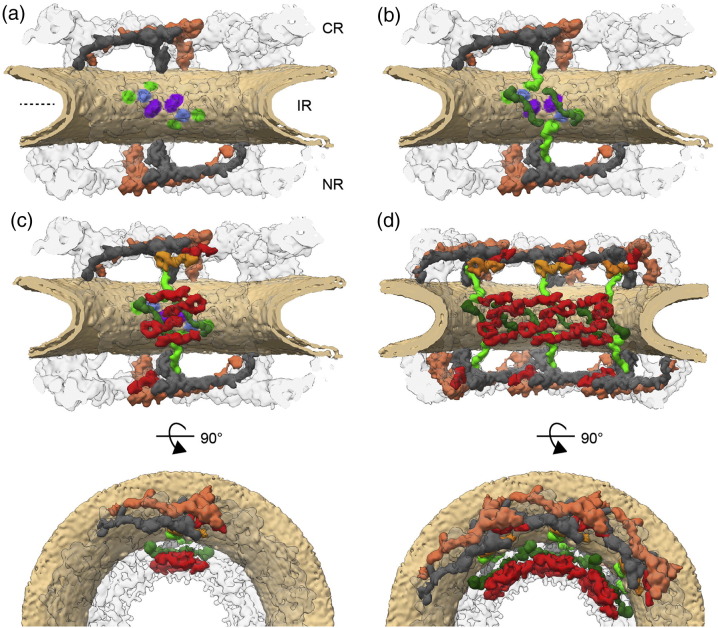
Scaffold architecture of the NPC [51]. The NPC is shown cut in half (a) four copies of the Y-complex per asymmetric unit of the NPC builds the scaffold of the cytoplasmic ring (CR) and the nucleoplasmic ring (NR). The outer copy is shown in orange; the inner copy is shown in gray. Multiple membrane contacts are apparent in the inner ring structure: density that contacts the outer leaf of the bilipid layer is shown in green and purple; apparent transmembrane domains are shown in blue. (b) Nup155 (green) appears to interact with the membrane at the points indicated in (a). (c) Six question mark densities (red) per asymmetric unit resembling the shapes of Nup205 or Nup188 localize to the CR, IR and NR. Another copy might reside only on the cytoplasmic site and is shown in orange. (d) Proteins of neighboring asymmetric units are shown as well.

Although the *in situ* arrangement of the Y-complex is relatively well understood, our understanding of inner ring architecture remains underdeveloped. However, based on systematic fitting of inner ring structures into the tomographic maps and biochemical data, a few principles about the architecture of the inner ring might be proposed. The tomographic map of the human NPC clearly resolves the lipid bilayer of the membrane. Also, regions where it has been displaced by other components are apparent, which give valuable insights about how the NPC scaffold is anchored to the NE [51]. Out of the three integral membrane proteins, only Ndc1 has a transmembrane domain that is large enough to be detected at this resolution [51], while Pom121 and gp210 cannot be resolved. Thus, one might speculate that Ndc1 occupies a *C*2-symmetric pair of transmembrane spanning densities, which are apparent in the inner ring region close to the NE symmetry plane (Fig. 4a). This potential localization of Ndc1 might be considered as an anchor point for scaffold Nups. In close proximity, two membrane touching points of the scaffold structure into which the systematic fitting approach has placed the membrane binding beta propeller of Nup155 are apparent (Fig. 4a and b). A proximity to Ndc1 is in line with biochemical interaction data [11]. However, a third membrane touching point is apparent in the respective region, which might be attributed to the membrane binding protein Nup35, which plays a crucial role in inner ring assembly and has been shown to engage with Nup155 and Ndc1 [23], [62], [63], [64]. Nup35 dimerizes and thus might direct Nup155 in a pairwise fashion at the site of Ndc1. In this model, two pairs of Nup155 point their alpha solenoid region into two opposing directions, thereby crossing the entire inner ring in an antiparallel fashion on the one hand and bridging it to the outer rings on the other hand. Nup155 could thus function as a spacer to maintain the distance to the outer rings. Interestingly, it has been experimentally demonstrated or predicted, respectively, that the beta propeller domains of the Y-complex members Nup133 and Nup160 interact with the NE membrane *via* an amphipathic alpha helical membrane binding motif [65], [66]. These two proteins are structurally related to Nup155. In all three cases, their fits in to the tomographic map suggest that their membrane binding motif dips into the outer leaf of the lipid bilayer but does not entirely penetrate it *in situ* [51].

The inner ring complex also comprises Nup93, Nup205 and Nup188 [11]. While Nup205 and Nup188 are large proteins with a very characteristic question mark shape [67], [68] that can be fitted in the tomographic map, Nup93 is too small. However, Nup205 and Nup188 are paralogous and cannot be distinguished from each other at the current resolution [51]. The systematic fitting approach suggested that 32 copies of these proteins symmetrically locate into the inner ring in a staggered fashion close to Nup155 and as a second layer that is more distant from the membrane (Fig. 4c and d). In addition to these inner ring locations, additional hits are obtained in close proximity of the Y-complex vertices within the outer rings [51], suggesting that additional 16 copies of Nup205/Nup188 might locate into the CR but only 8 copies might locate into the NR (Fig. 4c). None of the suggested location interferes with already assigned density. Biochemical evidence supports binding of Nup205 and Nup188 to the outer rings: first, Nup188 and Nup93 bind to the cytoplasmic Nup214–Nup84–Nup62–Nup98–Rae1 complex [51], which was is further supported by cross-linking mass spectrometric data [7]. Second, affinity isolations of Nup205 copurified Y-complex members. Higher-resolved cryoEM maps and further biochemical analysis are needed to clarify how exactly Nup205 and 188 distribute within the scaffold and to understand the inner ring architecture in more detail.

## Outlook

The analysis of the nuclear pore structure *in situ* has recently gained momentum, to some extent owing to technical advances in cryoEM but obviously also due to various other advances. Recent studies have obtained a resolution that is sufficient to interpret the 3D EM maps obtained from an *in situ* scenario in the context of biochemical and structural data obtained *in vitro*. These studies revealed that the NPC structure is even more complex than originally anticipated. In example, it has turned out that Nups cannot be assigned to only one subcomplex as shown for Nup188 and Nup93 that bind the cytoplasmic Nup214–Nup84–Nup62–Nup98–Rae1 complex and Nup62 that assembles with Nup58–Nup54 and additionally with Nup188–Nup93–Nup214–Nup84–Nup98–Rae1 [13], [14], [51]. The fact that multiple copies of the same protein might engage in different local interactions within the NPC assembly complicates the interpretation of various biochemical and interaction data. Many open questions regarding the structure of the NPC remain to be answered. How exactly is the inner ring complex built and held together? Do the nuclear-ring-specific Nups such as TPR, Nup153 and Nup50 contribute to NPC scaffold formation and if so, how?

A thorough understanding of NPC structure would enable further functional investigations and is therefore of high human health relevance [69], [70]. In light of the vast amount of experimental data available for lower eukaryotes, it appears very important to investigate possible differences of the NPC structure across the tree of life. Insights into NPC structure of lower eukaryotes might not only reveal the evolutionary principles but also give an idea about the similarity of the structures. Advances in automated data acquisition, data processing and technological breakthroughs such as direct electron detection have not yet been exploited to investigate the lower eukaryotic structure but hold great potential to clarify this point. The combination with biochemical and cell biological approaches and X-ray crystallography will likely be a powerful tool to answer these and related questions concerning the NPC structure as a joint effort of the scientific field.
